# Archival biogenic micro- and nanostructure data analysis: Signatures of diagenetic systems

**DOI:** 10.1016/j.dib.2018.05.041

**Published:** 2018-05-19

**Authors:** Laura A. Casella, María del Mar Simonet Roda, Lucia Angiolini, Andreas Ziegler, Wolfgang W. Schmahl, Uwe Brand, Erika Griesshaber

**Affiliations:** a Department of Earth and Environmental Sciences, Ludwig-Maximilians-Universität München, Theresienstr. 41, 80333 Munich, Germany; b Dipartimento di Scienze della Terra “A. Desio”, Università degli Studi di Milano, Via Mangiagalli 34, 20133 Milan, Italy; c Central Facility for Electron Microscopy, University of Ulm, Albert-Einstein-Allee 11, 89069 Ulm, Germany; d Department of Earth Sciences, Brock University, 1812 Sir Isaac Brock Way, St. Catharines, Ontario, Canada L2S 3S1

**Keywords:** Nanocomposite mesocrystal biocarbonate [NMB], Inorganic rhombohedral calcite [IRC], Diagenetic tracers: FE-SEM, AFM, EBSD, Brachiopods, Diagenetic alteration, Low-Mg calcite

## Abstract

The present data in brief article provides additional data and information to our research article “Micro- and nanostructures reflect the degree of diagenetic alteration in modern and fossil brachiopod shell calcite: a multi-analytical screening approach (CL, FE-SEM, AFM, EBSD)” [1] (Casella et al.). We present fibre morphology, nano- and microstructure, as well as calcite crystal orientations and textures found in pristine, experimentally altered (hydrothermal and thermal), and diagenetically overprinted brachiopod shells. Combination of the screening tools AFM, FE-SEM, and EBSD allows to observe a significant change in microstructural and textural features with an increasing degree of laboratory-based and naturally occurring diagenetic alteration. Amalgamation of neighbouring fibres was observed on the micrometre scale level, whereas progressive decomposition of biopolymers in the shells and fusion of nanoparticulate calcite crystals was detected on the nanometre scale. The presented data in this article and the study described in [1] allows for qualitative information on the degree of diagenetic alteration of fossil archives used for palaeoclimate reconstruction.

**Specifications Table**

**Table:** 

Subject area	*Crystallography*
More specific subject area	*Micro- and nanostructure of modern and fossil biogenic carbonate archives*
Type of data	*Figures, text file*
How data was acquired	*Microtome: Leica Ultracut equipped with glass knives and DiATOME diamond knife*
	*Critical Point Drying: BAL-TEC CPD 030*
	*FE-SEM: Hitachi S5200 field emission SEM*
	*EBSD: Hitachi SU5000 field emission SEM equipped with a Nordlys II EBSD detector and AZTec acquisition software*
	*AFM: JPK Instruments NanoWizard II equipped with a n*^*+*^*-silicon cantilever, measurements were conducted in contact mode*
Data format	*Analysed*
Experimental factors	*Thermal and hydrothermal alteration experiments*
Experimental features	*Thermal alteration experiments on modern brachiopod specimens were performed under dry conditions at 100 °C (for 72 hours, and three months), and at 400 °C (for 48 hours).*
	*Hydrothermal alteration experiments on modern brachiopod specimens were conducted in either simulated meteoric or burial fluids at 175 °C for 28 days.*
	*Pristine, thermally and hydrothermally altered, and fossil brachiopod shell fragments were embedded in epoxy resin and successively ground and polished for further investigations [see 1, 2].*
Data source location	*Friday Harbor Laboratories, University of Washington, U.S.A. (Terebratalia transversa),*
	*Signy and Rothera Islands, Antarctica (Liothyrella uva),*
	*Upper Ordovician Dillsboro Formation, Indiana, U.S.A (Platystrophia laticostata), Lower Jurassic Ait Athmane Formation of the Central High Atlas Basin, Morocco (Quadratirhynchia attenuata) ,*
	*Luc-Sur-Mer, Normandy, France (Digonella digona) and, Bakony Mountains, Hungary (Lobothyris punctata).*
Data accessibility	*Data is with this article*

**Value of the data**

• The data provides fundamental, quantitative and qualitative information on the assessment of the degree of (diagenetic) alteration of brachiopod shells.
• Hydrothermal alteration experiments mimicking diagenetic alteration may be applied to other biogenic hard tissues and inorganic mineral assemblages (e.g., rocks) in order to objectively compare the degree of diagenetic overprint.
• Data analysed by multi-analytical screening methods (FE-SEM, EBSD, AFM) may provide crucial information on the history of fossils used in research fields such as reconstruction of the palaeoclimates and palaeoenvironments.
• A comparison between microstructure and texture analyses of our data with isotope analysis may provide more detailed understanding of diagenetic overprint of fossil samples.

## 1 Data

**Fig. 1:**
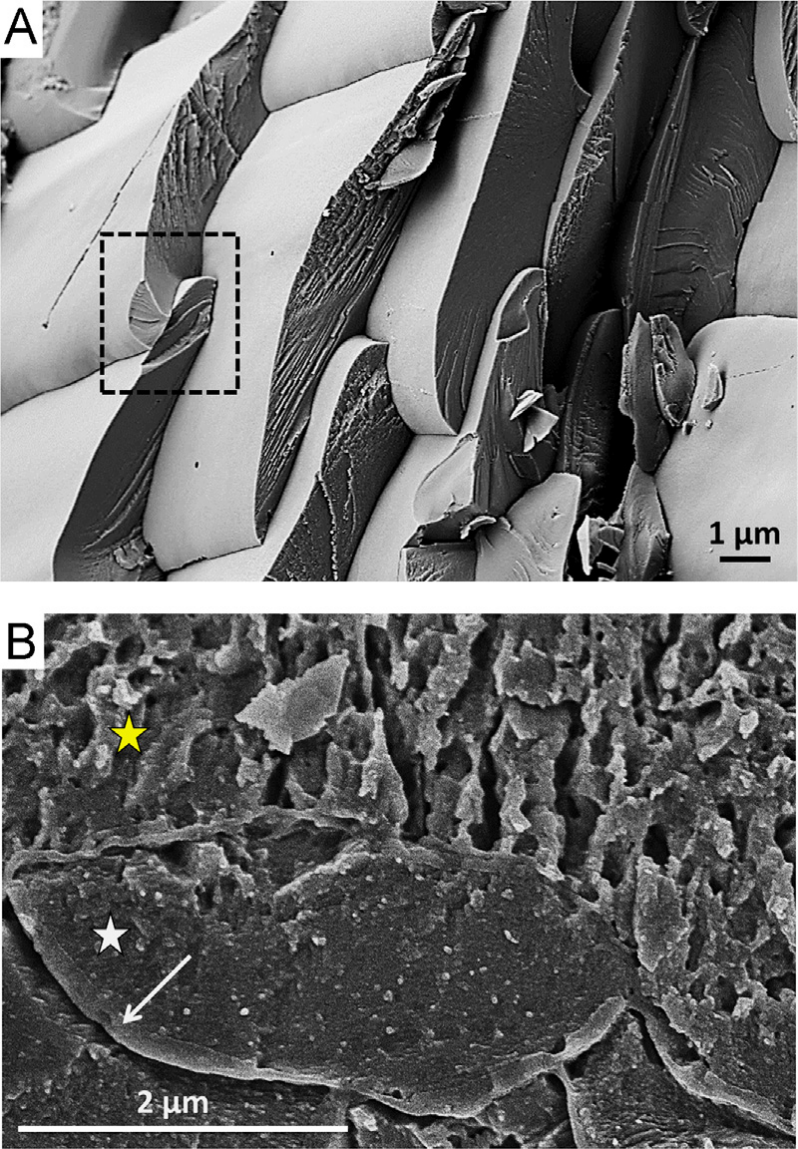
FE-SEM images of pristine *T. transversa* showing different fibre characteristics. (A) Fibres of the fibrous secondary layer exhibit uneven surfaces after mechanical fracturing (black dashed rectangle). (B) Irregularly shaped small mineral units as well as organic matrices can be observed in the primary layer (yellow star) of the *T. transversa* shell. Each single fibre of the fibrous secondary layer is surrounded by a biopolymer matrix (white arrow), and is comprised of nanoscopic biocomposite mesocrystals (white star).

**Fig. 2:**
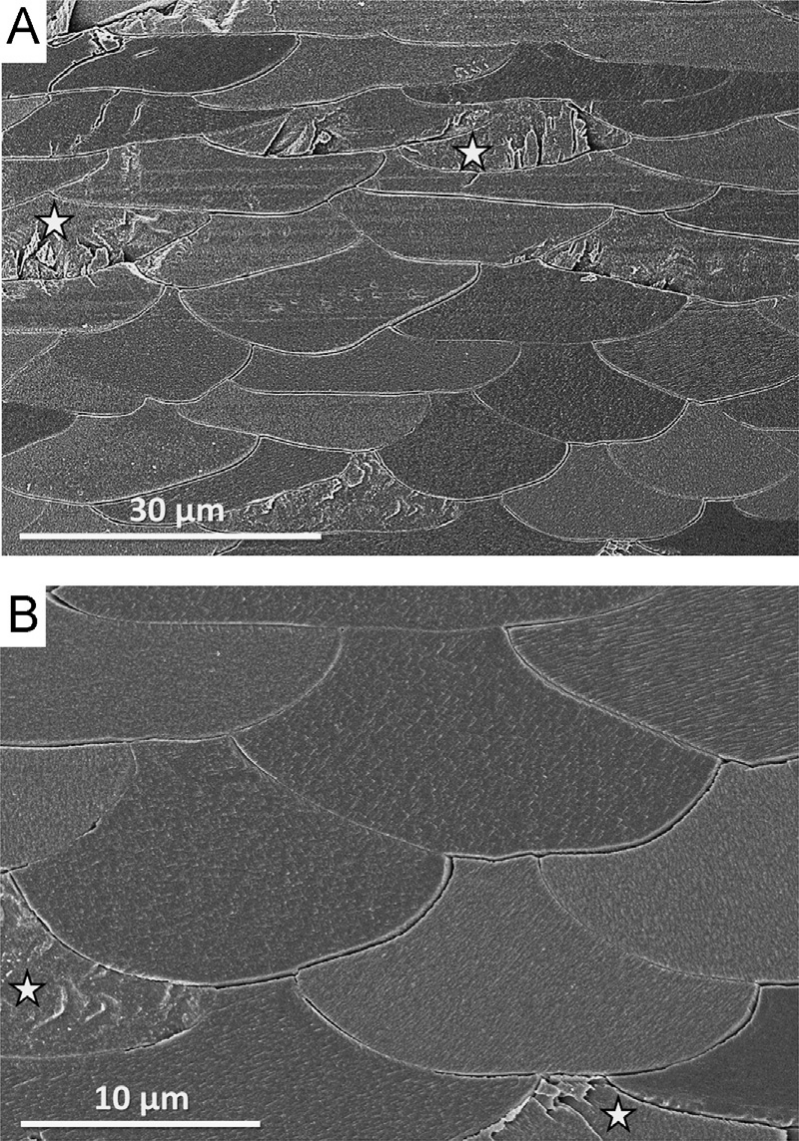
FE-SEM images of microtome cut, polished, etched and critical point dried surfaces of the fossil brachiopod *Q. attenuata*. As the fossil shell is devoid of organics (membranes around the fibres, network of fibrils within the fibres), it becomes brittle and fractures when cut with a microtome knife (white stars in A and B).

**Fig. 3:**
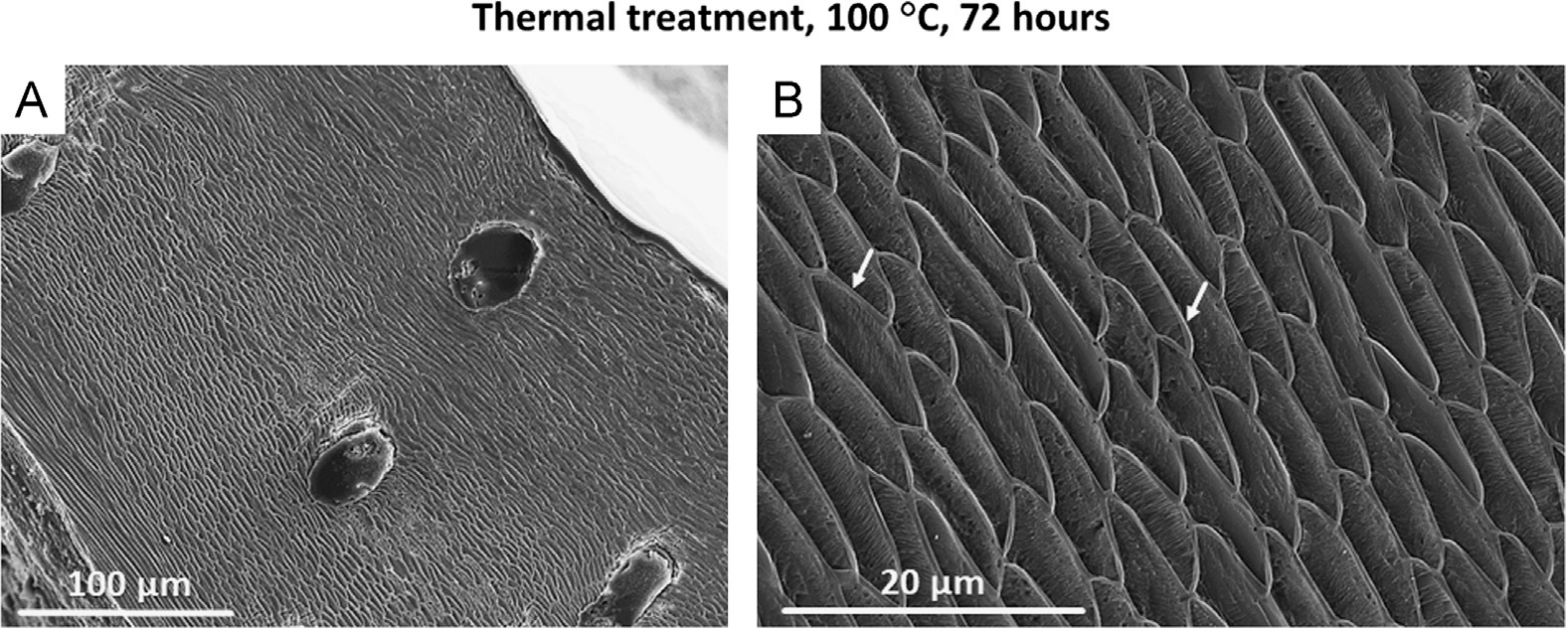
FE-SEM images of *L. uva* thermally altered under dry conditions at 100 °C for 72 hours. The morphology and arrays of fibres are well kept (A), and each fibre is surrounded by an organic membrane (white arrows in B).

**Fig. 4:**
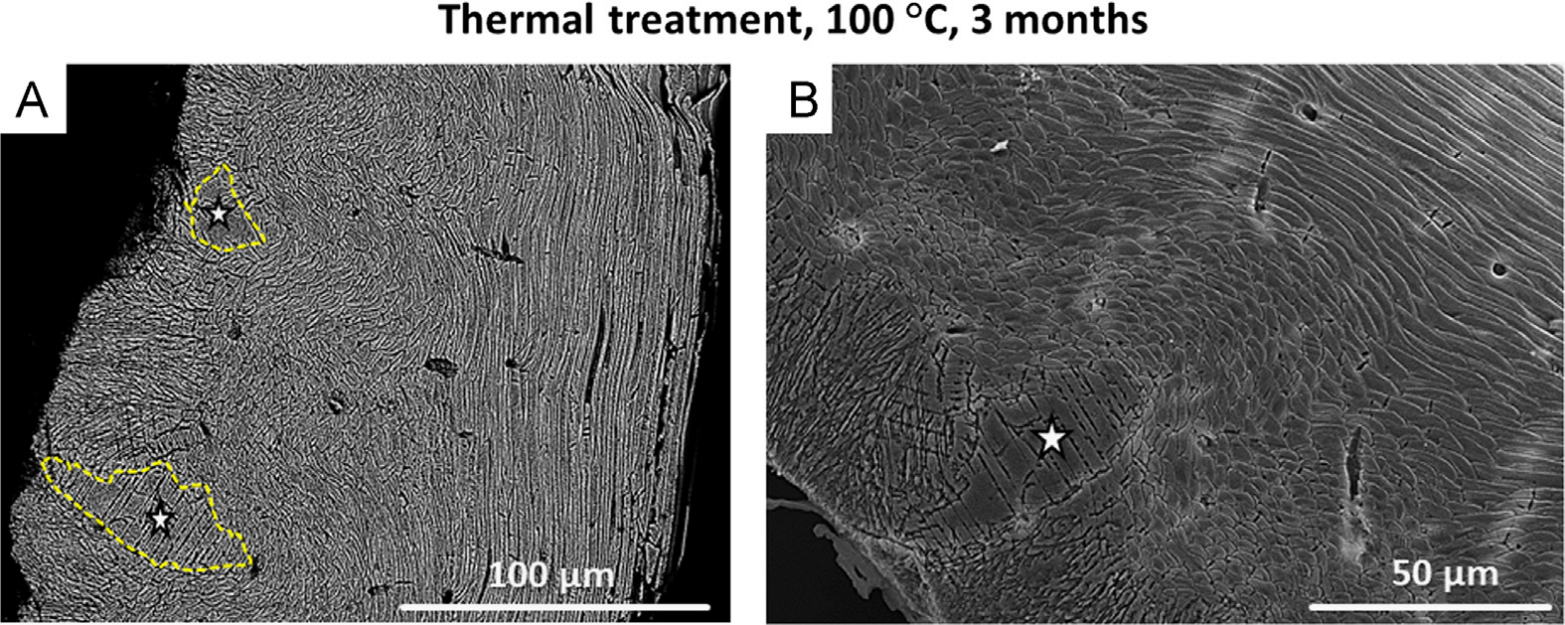
FE-SEM images of *L. uva* shells thermally altered under dry conditions at 100 °C for three months. New, large, irregularly shaped calcite units developed (white stars in A and B, yellow dashed outlines in A) as the original basic mineral units of the skeleton amalgamate.

**Fig. 5:**
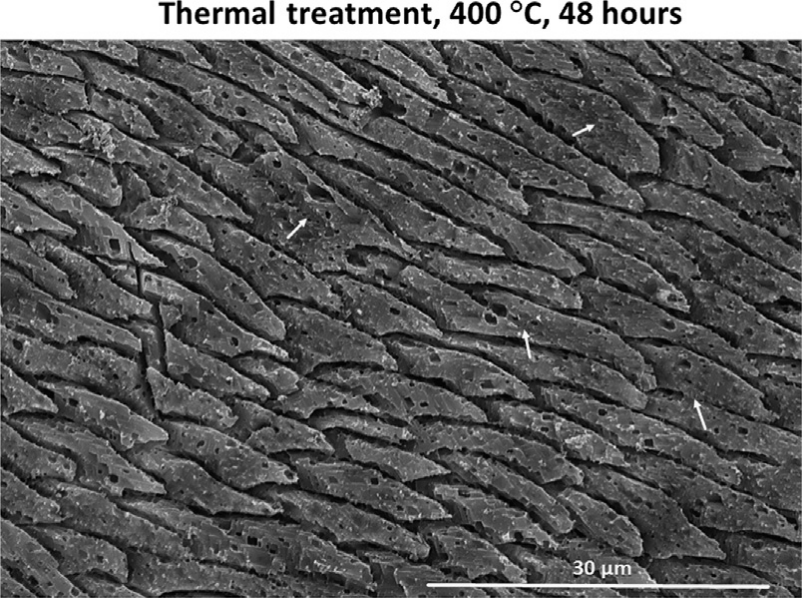
FE-SEM image of a *L. uva* shell thermally treated at 400 °C for 48 hours. Organic membranes are entirely decomposed; new calcite formation starts to occur as neighbouring fibres amalgamate (white arrows).

**Fig. 6:**
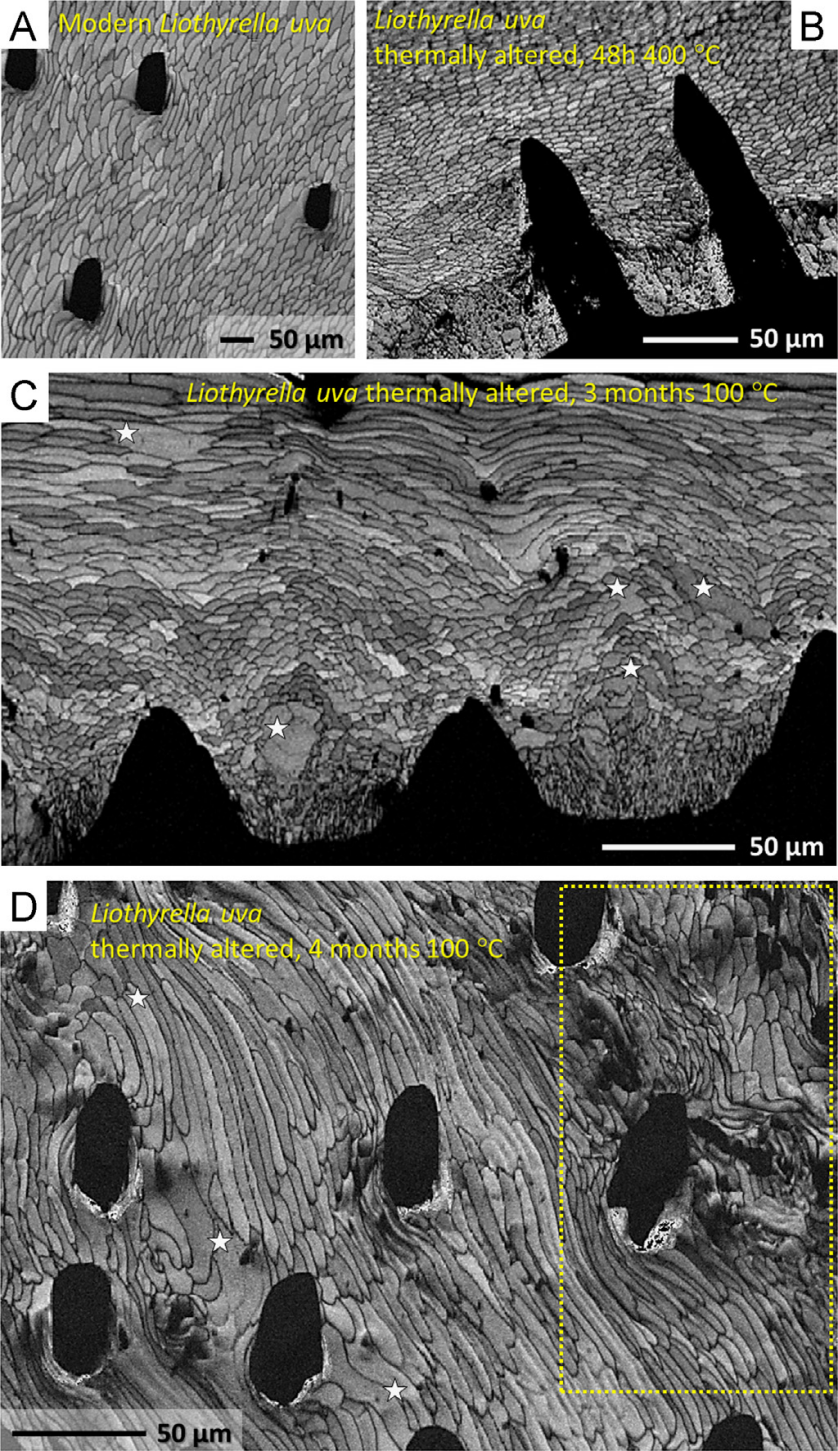
EBSD band contrast images of pristine and thermally altered *L. uva* shells show the change and distortion of microstructure of the fibrous layer with progressive alteration times and temperatures. Relative to pristine *L. uva* (A), a slight distortion of the microstructure can be observed in shell samples altered at 400 °C and for 48 h (B). New mineral formation and fibre amalgamation was observed after thermal alteration for three months at 100 °C (white stars in C). Alteration for four months at 100 °C caused significant fibre amalgamation (white stars in D), microstructure destruction (yellow dashed rectangle in D) and new calcite formation.

**Fig. 7:**
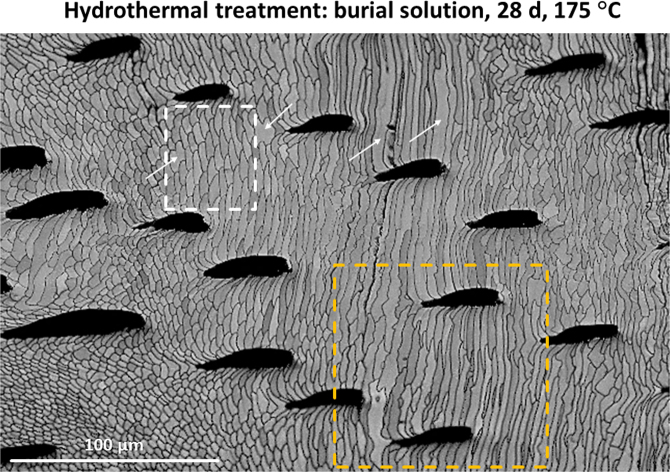
EBSD band contrast image of hydrothermally altered *T. transversa* shell. Alteration occurred at 175 °C for 28 days and was carried out in simulated burial fluid. Shell areas where the original fibre morphology was distorted by alteration (yellow rectangle) can be observed close to regions where the shell microstructure was little affected (white rectangle). Note that amalgamation of fibres occurred occasionally (white arrows).

**Fig. 8:**
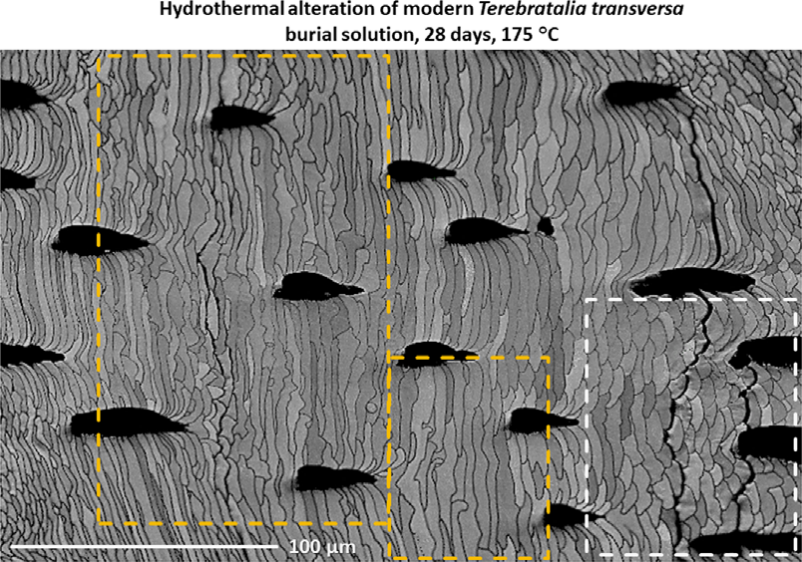
EBSD band contrast image of hydrothermally altered *T. transversa* shell. Alteration occurred at 175 °C for 28 days and was carried out in simulated burial fluid. In some parts of the shell the original fibrous shell microstructure was distorted by amalgamation of fibres due to alteration (yellow dashed rectangles). The amalgamation of fibres can be explained by lateral growth of the inorganic rhombohedral calcite (IRC) of one nanocomposite mesocrystal biocarbonate (NMB) fibre growing into the neighbouring fibre. Note that altered shell areas are next to shell areas which appear to be little affected by alteration (white dashed rectangle).

**Fig. 9:**
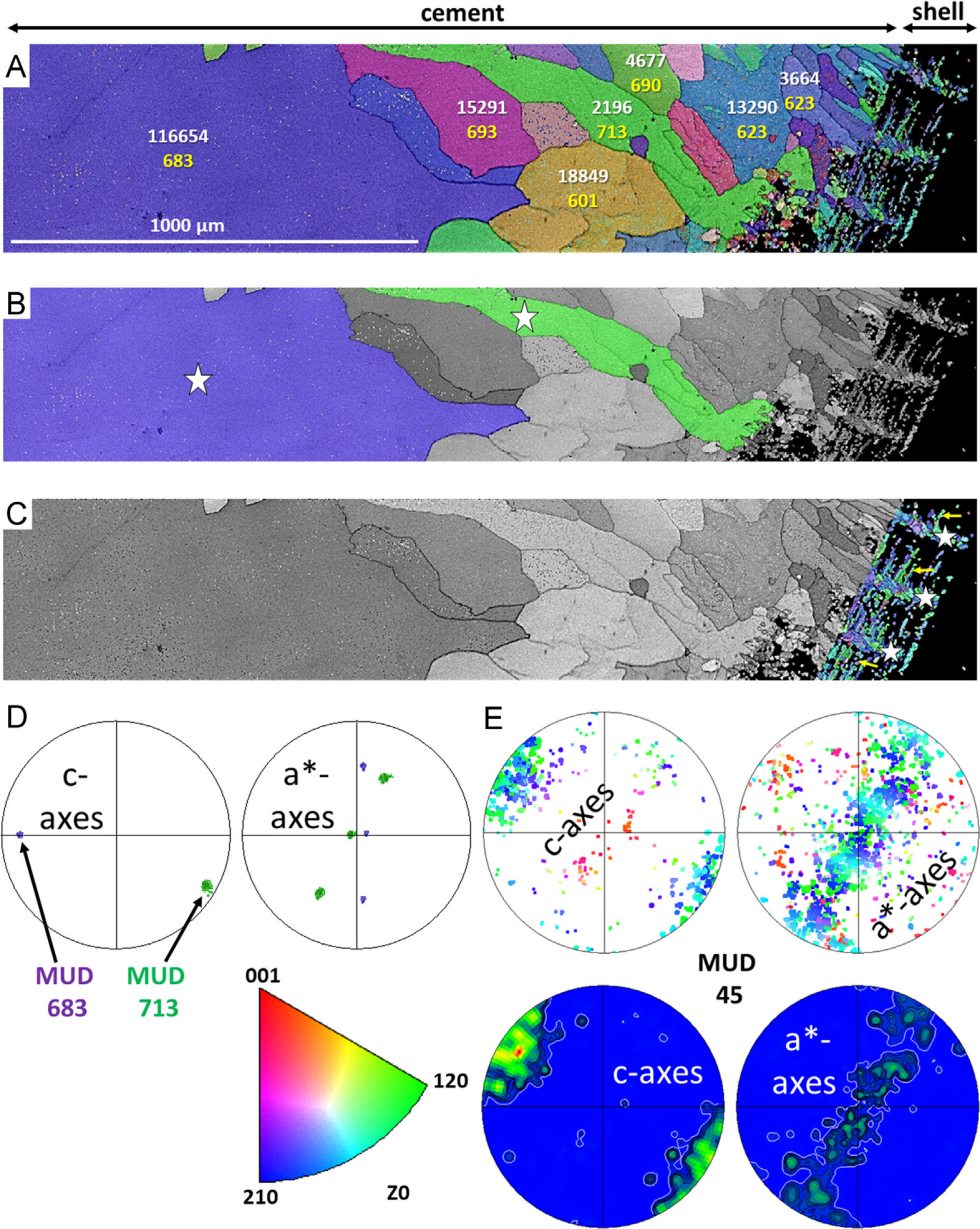
EBSD band contrast images and colour-coded orientation maps with corresponding pole figures of fossil brachiopod *D. digona* (A, C, E) and cement found between the pedicle and brachial valves (A, B, D; see Fig. 10D in [1]). Recrystallised calcite is present in the fossil *D. digona* shell (white stars in C) and as precipitate within endopunctae (yellow arrows in C) and cement (white stars in B). (A) Yellow numbers show MUD values of individual calcite grains found within the cement. The number of corresponding data points used for the calculation of each MUD value is given in white. (D) MUD values of two selected recrystallised calcite grains of the cement (coloured, white stars in B) are similar to each other (683 and 713) and show characteristics of a single crystalline phase due to the superposition of crystallographic axes on the pole figures. (E) In contrast, the crystallographic axes of newly formed calcite found within the shell of *D. digona* show high misorientation on the pole figures and, thus, a significantly lower MUD value of 45 is obtained.

**Fig. 10:**
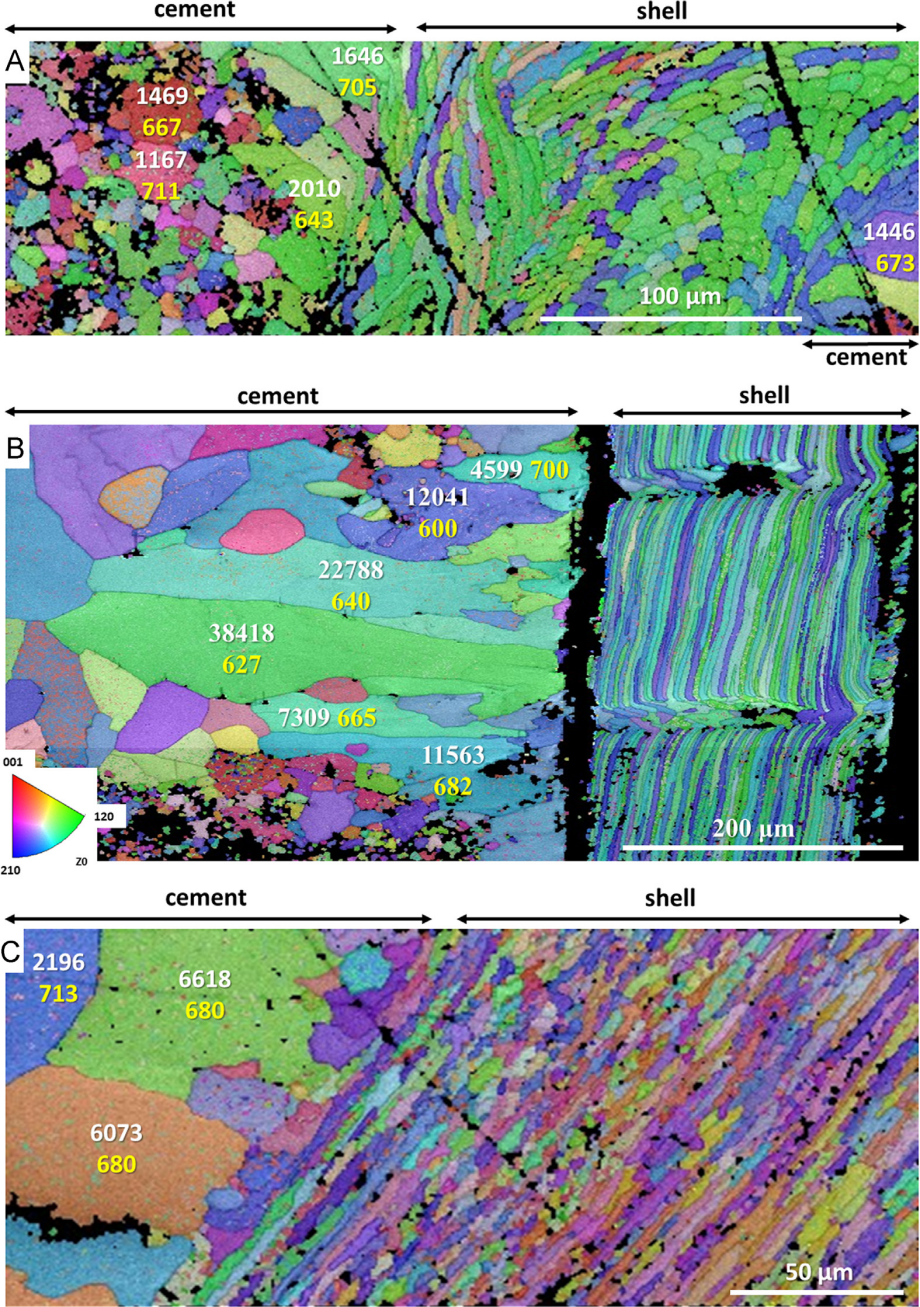
EBSD colour-coded orientation maps showing recrystallised calcite inside the shells of the fossil brachiopods *L. punctata* (A), *D. digona* (B) and *P. laticostata* (C). MUD values are given in yellow, the number of calcite data points within a large calcite crystal is shown in white.

**Fig. 11:**
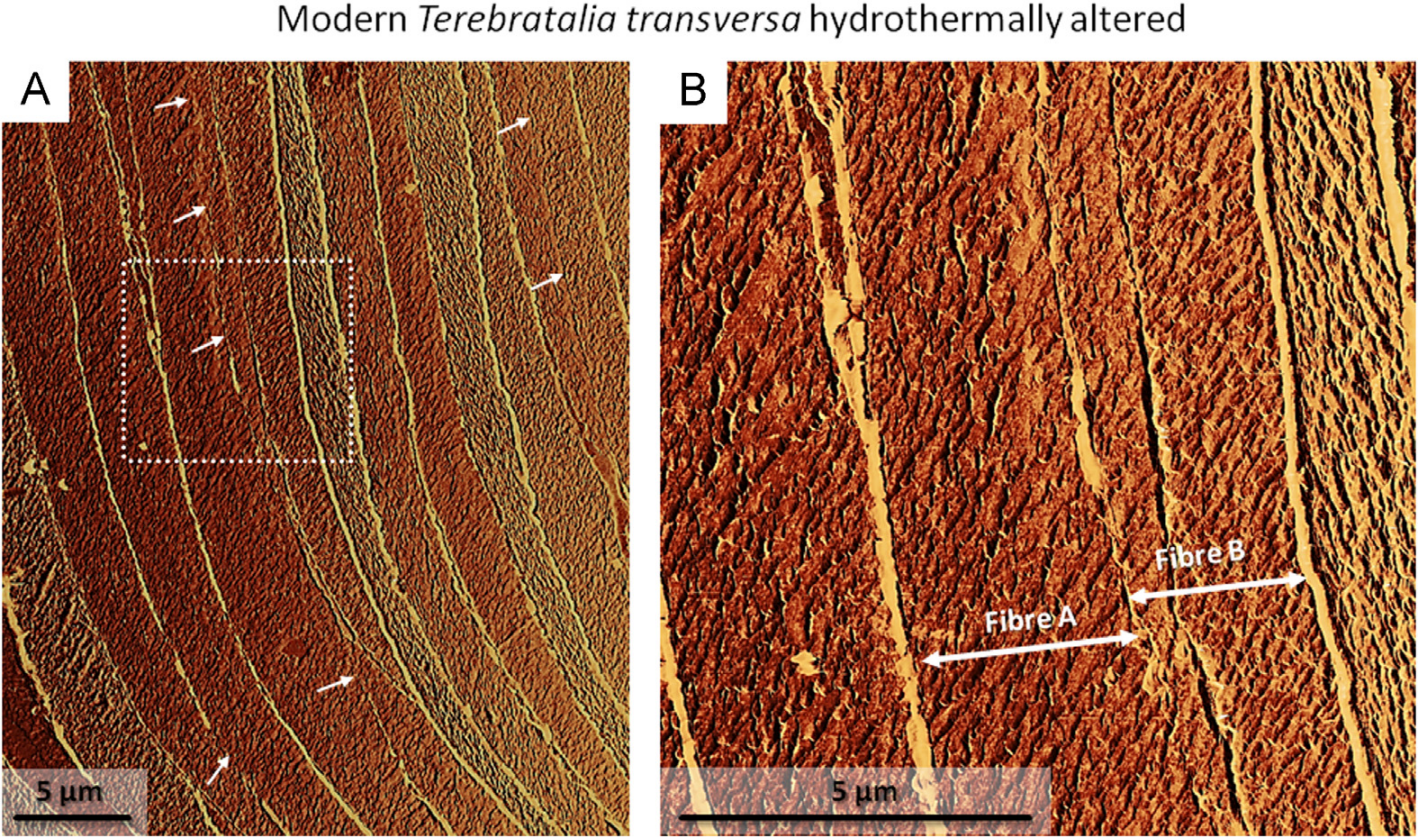
Lateral deflection AFM images of hydrothermally altered *T. transversa* shell pieces. Alteration was carried out in simulated meteoric fluid at 175 °C and lasted for 28 days. The degradation of organic membranes, the amalgamation of neighbouring fibres (white arrows in A), and new calcite formation (A, B) can be observed at sites of former membranes located between two fibres. The dashed white rectangle in (A) indicates the location of the shell area shown in (B).

**Fig. 12:**
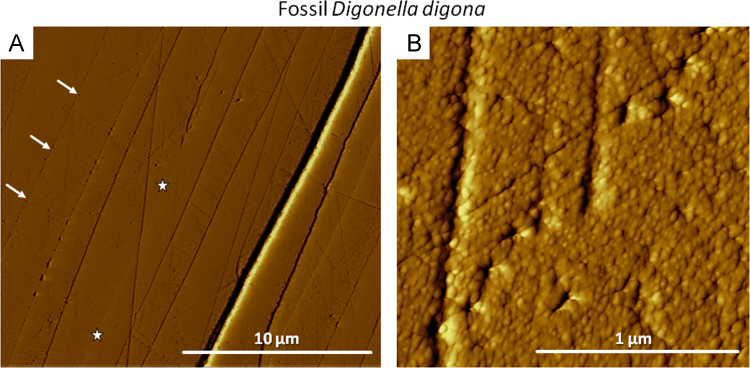
Vertical deflection AFM images of fossil *D. digona* shell pieces. Degradation of organic membranes (white arrows in A) and amalgamation of neighbouring fibres into larger units (white stars in A) can be observed in the Jurassic brachiopod specimen. (B) Formation of inorganic rhombohedral calcite (IRC) crystallites occurred within a single calcite fibre of the fibrous secondary layer.

**Fig. 13:**
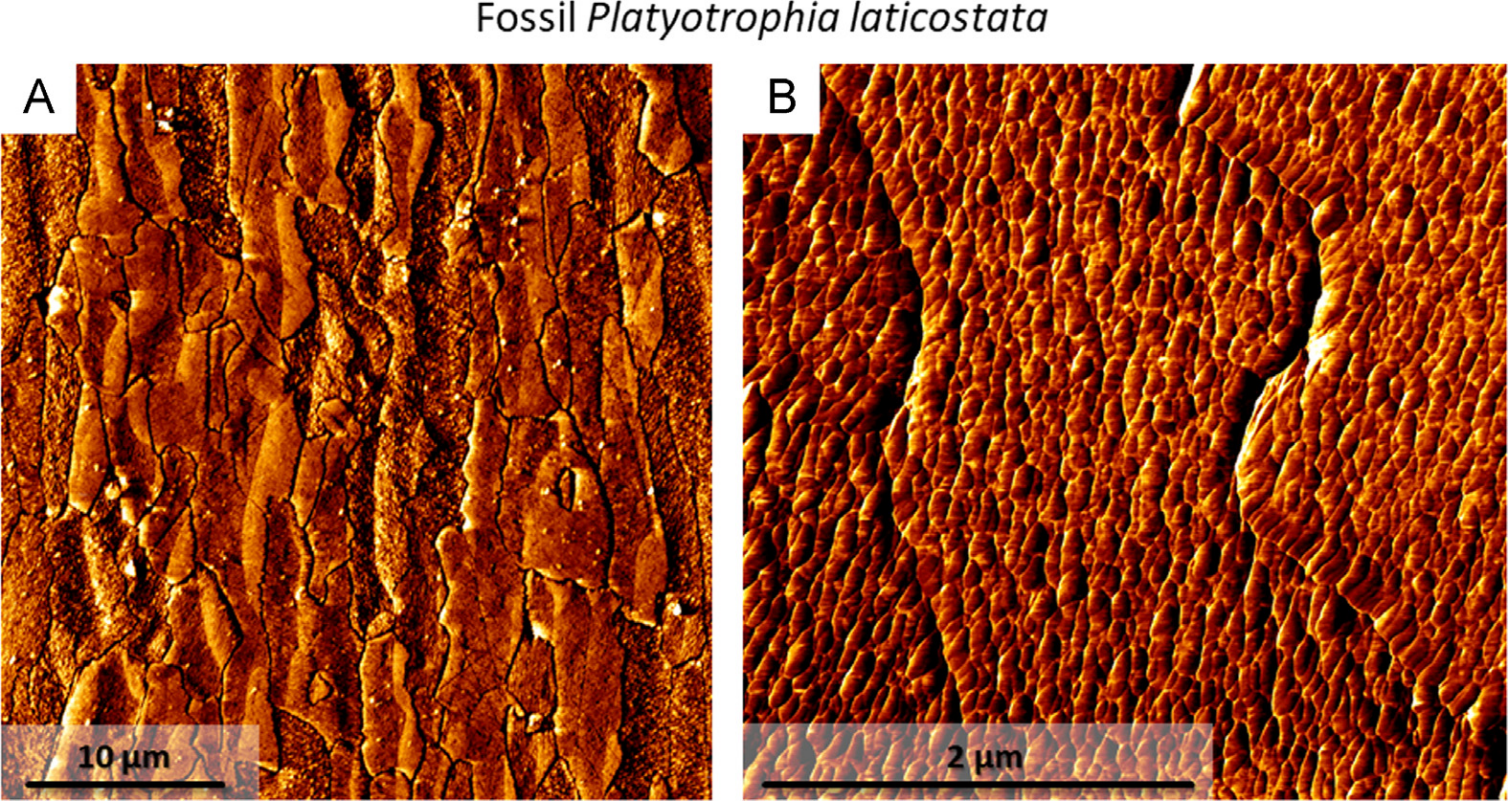
Lateral deflection AFM images of fossil *P. laticostata* shell pieces showing (A) the distortion of neighbouring calcite of the fibrous secondary layer caused by diagenetic overprint. (B) Fibres of fossil *P. laticostata* lack the enclosing organic membranes and consist of inorganic calcite crystallites.

Among marine biocarbonates, calcitic brachiopod shells are one of the most used archives in palaeoclimate and palaeoecological research [3], [4], [5], [6], [7], [8]. In the past few decades, basic knowledge on microstructural and textural patterns of pristine brachiopod shells was established [9], [10], [11], [12], [13], [14], [15], [16], [17], [18], [19], [20]. Here, we focus on additional insights on microstructural and nanostructural characteristics caused by (mimicked) diagenetic alteration by using biochemical etching (Fig. 1, Fig. 2), FE-SEM (Fig. 1, Fig. 2, Fig. 3, Fig. 4, Fig. 5), EBSD orientation and texture data evaluation (Fig. 6, Fig. 7, Fig. 8, Fig. 9, Fig. 10), and AFM imaging (Fig. 11, Fig. 12, Fig. 13) of pristine, thermally, hydrothermally, and diagenetically altered brachiopod shells. Based on FE-SEM imaging and EBSD measurements, high-resolution data on pristine, (hydro-) thermally altered, and fossil brachiopod specimens was obtained and subsequently analysed. We compare micro- and nanostructural data of pristine and (hydro-) thermally or diagenetically altered brachiopod shells, i.e., the presence of organic matrices, and the shape of calcite fibres of the fibrous secondary shell layer. Texture analysis deduced from EBSD measurements on fossil brachiopods with varying degrees of diagenetic overprint shows the relation between the degree of crystallographic co-orientation and diagenetic history experienced by biogenic minerals. AFM imaging of hydrothermally and fossil brachiopod shells provides supporting and more detailed data on fibre morphologies and their internal structure.

## 2 Experimental design, materials and methods

### 2.1 Experimental designs of simulated diagenetic alteration

#### 2.1.1 Thermal alteration

Thermal alteration was carried out in order to observe the decomposition of the enclosing organic membranes. Sample material used in thermal alteration experiments was heated in an oven at 100 °C for 72 h and for three months, as well as at 400 °C for 48 h.

#### 2.1.2 Hydrothermal alteration

Hydrothermal alteration experiments were performed in the presence of either simulated meteoric (10 mM NaCl aqueous solution) or simulated burial (100 mM NaCl + 10 mM MgCl_2_ aqueous solution) fluid. Both solutions were prepared using high-purity deionised water [1], [2], [21]. Sample material and 10 ml of fluid were inserted into a polytetrafluoroethylene crucible which was placed inside a metal autoclave. Experiments were conducted at 100 °C for 28 days using either simulated meteoric or burial fluid. Pressure conditions during the hydrothermal alteration experiments corresponded to the vapour pressure of water at the given temperature.

### 2.2 Pristine and fossil brachiopod materials

#### 2.2.1 Investigated pristine brachiopods

Modern brachiopod specimens of *Terebratalia transversa* (Sowerby, 1846) and *Liothyrella uva* (Broderip, 1833) were utilised in biochemical studies for establishing baselines of pristine brachiopod shell micro- and nanostructures, as well as in thermal and hydrothermal alteration experiments mimicking diagenetic alteration of biogenic carbonates. Sampling sites of both live collected brachiopods were Friday Harbor Laboratories, University of Washington, U.S.A., and Signy and Rothera Islands, Antarctica, for *T. transversa* and *L. uva*, respectively.

#### 2.2.2 Investigated fossil brachiopods

Four fossil equivalents were chosen from basins which experienced different burial depths and diagenetic temperatures. *Platystrophia laticostata* (James, 1871) was collected from the Upper Ordovician Dillsboro Formation, Indiana, U.S.A. The Jurassic brachiopods *Lobothyris punctata* (Sowerby, 1812) and *Quadratirhynchia attenuata* (Dubar, 1931) were collected at the Bakony Mountains, Hungary, and Ait Athmane Formation of the Central Atlas Basin, Morocco, respectively. *Digonella digona* (Sowerby, 1815) is the youngest of all Jurassic brachiopod samples and its sampling site was located at Luc-Sur-Mer, Normandy, France. Further information on all utilised brachiopod specimens is given in [1].

### 2.3 Microtome cutting and polishing

Brachiopod shell fragments of pristine and fossil brachiopod species were mounted on 3 mm thick cylindrical aluminium rods using super glue. In order to obtain plane surfaces, the samples were microtome cut using a Leica Ultracut ultramicrotome equipped with glass knives. Subsequently, the cut specimens were polished by stepwise removal of material in a series of slices with successively decreasing thicknesses (90 nm, 70 nm, 40 nm, 20 nm, 10 nm, and 5 nm) using a DiATOME diamond knife. Each step was repeated 15 times [22].

### 2.4 Selective biochemical etching

Microtome-polished shell surfaces were etched and the organic matter fixed, simultaneously, while immersed in a solution of 0.1 M HEPES (pH= 6.5) and 2.5% glutaraldehyde for 180 seconds. The etching procedure was stopped by a dehydration step in 100% isopropyl 3 times for 10 minutes each. Subsequently, specimens were critical point dried using a BAL-TEC CPD 030 (Liechtenstein) device and rotary coated with 3 nm of platinum.

### 2.5 FE-SEM imaging

For FE-SEM imaging, selected sample material was prepared by microtome cutting and polishing following a selective biochemical etching treatment. However, the major preparation technique for SEM imaging and EBSD measurements of brachiopod samples was carried out as follows. Brachiopod shells were longitudinally cut from the umbo to the commissure and, subsequently, embedded in epoxy resin. Shell surfaces were conventionally ground and polished in sequential steps down to a grain size of 1 µm (particle size of polishing agent). The preparation was finalised by an etch-polishing step utilising colloidal alumina with particle sizes of approx. 0.05 µm in a vibratory polisher. Subsequently, sample surfaces were rotary coated with 4–6 nm of carbon.

FE-SEM imaging was carried out at 4, 5, or 10 kV using a Hitachi S5200 electron microscope.

### 2.6 EBSD measurements, band contrast and analysis of calcite orientation data

For EBSD measurements, brachiopod shells were conventionally ground and polished as described above. Sample surfaces were rotary coated with 4–6 nm of carbon.

EBSD measurements were conducted at 20 kV using a Hitachi SU5000 FE-SEM equipped with a Nordlys II EBSD detector and AZTec acquisition. Obtained EBSD data was evaluated using CHANNEL 5 HKL evaluation software [23], [24].

Data on crystal orientation is shown as band contrast images and colour-coded crystal orientation maps with corresponding pole figures. EBSD band contrast represents the quality of the Kikuchi diffraction pattern in each measured point, thus, strong EBSD signals result in a bright image point and weak or absent signals (e.g., due to pores, organic matter, amorphous phases) result in dark image points. Crystallographic orientation maps were derived from EBSD scans. A measure of co-orientation within single crystals or ensembles of crystals are given by the multiple of uniform distribution (MUD). High MUD values indicate highly co-oriented crystallographic axes (e.g., MUD of > 700 in inorganic single crystals [25]) and, thus, a strong texture. Low MUD values down to 1.0 reflect randomly oriented crystallographic axes, thus, a weak or no texture.

### 2.7 AFM imaging

For AFM imaging, brachiopod specimens were prepared by two different preparation techniques, i.e., microtome cutting using glass knives and polishing using a diamond knife, as well as by conventional grinding and polishing as is described above (see subchapters 2.3 and 2.5). The latter includes an additional step, i.e., cleaning of the highly polished sample surface for 10 min. using high-purity deionised water in an ultrasonic bath, rinsing with ethanol and subsequent air drying. Rotary coating was not applied prior to AFM imaging.

Atomic force microscopy was conducted on hydrothermally altered and fossil brachiopod shells. Images were taken in contact mode.
